# A comparative study of analytical models of diffuse reflectance in homogeneous biological tissues: Gelatin-based phantoms and Monte Carlo experiments

**DOI:** 10.1002/jbio.202300536

**Published:** 2024-04-14

**Authors:** Anisha Bahl, Silvere Segaud, Yijing Xie, Jonathan Shapey, Mads S. Bergholt, Tom Vercauteren

**Affiliations:** 1 School of Biomedical Engineering & Imaging Sciences, King's College London, London, UK; 2 King's College Hospital, London, UK; 3 Department of Craniofacial Development and Stem Cell Biology, King's College London, Guy's Tower, Great Maze Pond, London, UK

**Keywords:** biological models, gelatin, imaging phantoms, Monte Carlo simulations, oxygen saturation

## Abstract

Information about tissue oxygen saturation (StO_2_) and other related important physiological parameters can be extracted from diffuse reflectance spectra measured through non-contact imaging. Three analytical optical reflectance models for homogeneous, semi-infinite, tissue have been proposed (Modified Beer–Lambert, Jacques 1999, Yudovsky 2009) but these have not been directly compared for tissue parameter extraction purposes. We compare these analytical models using Monte Carlo (MC) simulated diffuse reflectance spectra and controlled gelatin-based phantoms with measured diffuse reflectance spectra and known ground truth composition parameters. The Yudovsky model performed best against MC simulations and measured spectra of tissue phantoms in terms of goodness of fit and parameter extraction accuracy followed closely by Jacques' model. In this study, Yudovsky's model appeared most robust; however, our results demonstrated that both Yudovsky and Jacques models are suitable for modeling tissue that can be approximated as a single, homogeneous, semi-infinite slab.

**Figure:**
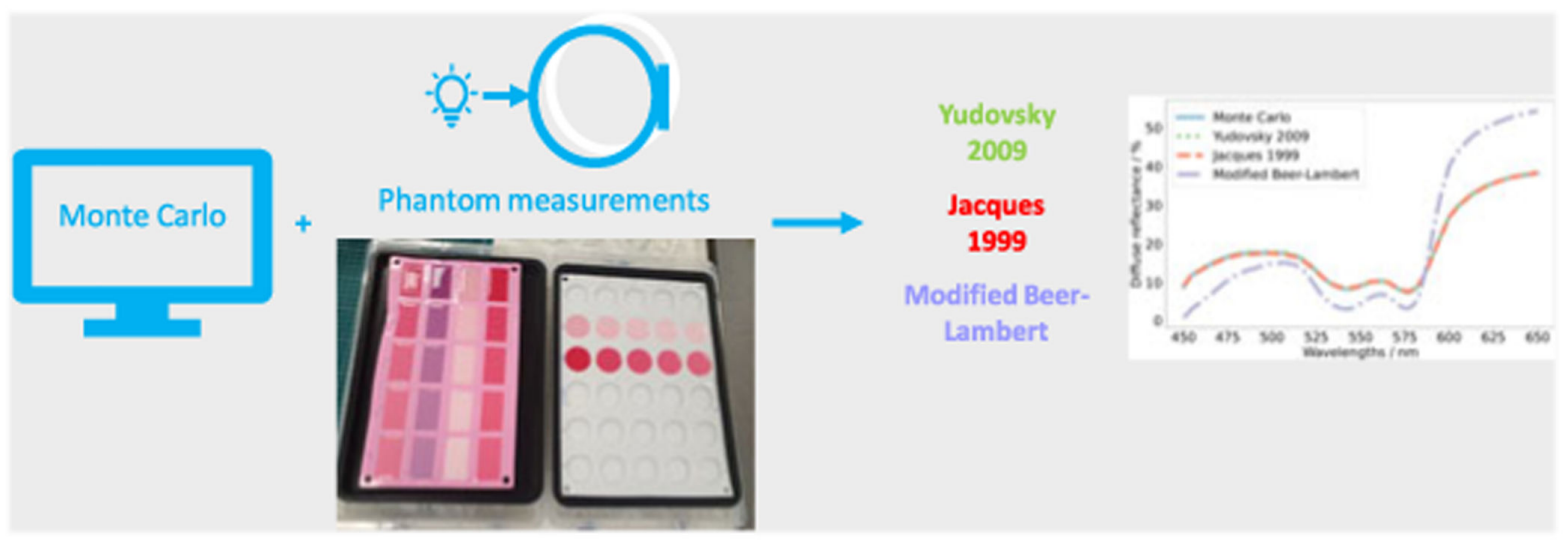

## 1 Introduction

Tissue oxygen saturation (StO_2_) is a key metric that could be used in surgery to determine tissue viability, tumor localization, and vessel flow [1–3]. There are, however, no currently established, spatially resolved, intra-operative methods of quantitatively determining StO_2_. In many clinical applications indocyanine green fluorescence angiography (ICG) is used to display perfusion as blood flow through vessels can be visualized by the fluorescence of the indocyanine dye given to the patient [4]. Whilst this method has shown clinical benefits [5, 6], it cannot quantify the StO_2_ of the tissues being visualized leading to subjective interpretation. There has been increasing promise that diffuses reflectance spectroscopic techniques could be used to obtain StO_2_ intra-operatively. Hyperspectral imaging (HSI) is one such technique that obtains a diffuse reflectance spectrum for each pixel of an image [7, 8], enabling intraoperative, non-contact, spatially resolved StO_2_ extraction without the need for a contrast agent.

There have been many proposed methods to extract StO_2_ parameters from tissue diffuse reflectance spectra ranging from the simplest ratiometric two-wavelength methods to more sophisticated analytical models [9] or deep-learning based approaches [10]. Using two or three wavelength models can provide good results but require data to be captured at these precise wavelengths placing large constraints on the measurement devices used and significant model assumptions. Analytical models can be applied to ranges of wavelengths for which they are developed and so these are favored in this work. Here we consider only visible range models (450–650 nm) as this allows for non-contact imaging using standard cold surgical light sources [11]. The visible light wavelength range also penetrates less deeply into tissue [12, 13]. Light–tissue interaction is therefore most likely to replicate a single homogeneous semi-infinite tissue layer and less likely to travel between layered structures. Tissue models tend to either be based on modifications of the Beer–Lambert model, or on the diffusion approximation particularly using the Kulbelka–Munk approximation method [9]. Three models that can be applied to homogeneous semi-infinite tissue in the visible wavelength range (450–650 nm) include (a) a modified Beer–Lambert model [14]; (b) a more elaborate Beer–Lambert-based model proposed by Jacques [15]; and (c) a semi-empirical model based on the Kulbelka–Munk theory as described by Yudovsky [16]. Alternatively, Monte Carlo (MC) methods may be used to model tissues for a range of wavelengths. While MC is an established approach for modelling light-tissue interaction, it is computationally challenging to apply in an inverse problem setting making the estimation of optical properties from tissue spectra non trivial on the basis of MC simulations alone. To address the need for improved inverse modelling utilizing MC, Inverse Adding Doubling (IAD) [17] has been proposed, however this is also slow iterative process and therefore not as efficient as approaches based on analytical models. A limitation of IAD is that, although a single reflectance measurement can be used to analyze semi-infinite samples with IAD, its primary focus is the extraction of optical properties from slabs of tissue with known thickness using both transmission and reflection measurements. In this work, IAD is used to provide information on optical properties from tissue phantom slabs but is not assessed in the same capacity as the other analytical models listed above.

Since there is no standard method of determining spatially resolved, ground truth, optical properties of tissues, simulations and physical phantoms must be used for testing purposes. Gelatin-based phantoms are commonly used to allow tunable absorption and scattering properties in solid phantoms [18, 19]. In this work we compare three major analytical models against both MC simulated spectra, and measured spectra from gelatin-based tissue phantoms with known ground-truth constituent quantities. These datasets cover a large parameter range mimicking that expected in biological tissue [20]. Gelatin phantoms are constructed to optically mimic biological tissue absorption and scattering ranges as closely as possible, while maintaining well-defined optical properties with known ground truth. We present a first direct comparison in the performance of these models against simulated and measured spectra, and aim to evaluate their potential for accurate StO_2_ extraction. This evaluation is quantified in terms of the forward models (utilizing ground truth values within the models to predict the diffuse reflectance spectra), and for the inverse problem solving (evaluating the fidelity of the parameter extraction when fitting to the simulated or measured data). Our results provide a comparison which can be used to inform the translation of these models into clinical applications.

## 2 Materials and Methods

### 2.1 Tissue models

Three analytical models are compared and evaluated in this work: modified Beer–Lambert [14], Jacques 1999 [15], and Yudovsky 2009 [16]. Due to the absorbing and scattering nature of biological tissue these models are used to analyze diffuse reflectance spectra which result from propagation of light via many optical paths through the tissue.

#### 2.1.1 Common absorption and scattering model

All three forward models use the wavelength dependent absorption and reduced scattering coefficients (*μ*_*a*_(*λ*) and $\mu_{s}^{\prime }(\lambda )$) of the tissue as inputs to compute a diffuse reflectance spectrum. The reduced scattering coefficient comprises of the scattering coefficient (*μ*_*s*_(*λ*)) and tissue anisotropy (*g*) as follows:

(1) $$\mu_{s}^{\prime }(\lambda )=\mu_{s}(\lambda )\times (1-g).$$

For biological tissue, the reduced scattering coefficient can be well approximated using Mie theory [20]:

(2) $$\mu_{s}^{\prime }(\lambda )=a{\left(\frac{\lambda }{500}\right)}^{-b},$$

where *a* and *b* are Mie scattering coefficients that range between 8 and 70 cm^−1^, and 0.1 and 3.3 respectively depending on the tissue microstructure [20]. The absorption coefficient of most internal, homogeneous, single-layer tissues is dominated by hemoglobin in the visible region (450–650 nm) [21] and can be modelled using the following equation [16]:

$$\mu_{a}(\lambda )=f_{\text{blood}}\mu_{a,\text{blood}}(\lambda )+(1-f_{\text{blood}})\mu_{a,\text{back}}(\lambda )$$

where

$$\mu_{a,\text{blood}}(\lambda )=c_{\text{HbT}}\frac{\ln(10)}{64500}[{\text{StO}}_{2}\varepsilon_{{\text{HbO}}_{2}}(\lambda )+(1-{\text{StO}}_{2})\varepsilon_{\text{Hb}}(\lambda )]$$

(3) $$\mu_{\text{a},\text{back}}(\lambda )=7.84\times 10^{8}\lambda^{-3.255}$$

Here StO_2_ refers to oxygen saturation, *c*_HbT_ refers to the total concentration of hemoglobin in whole blood commonly taken as 150 g L^−1^ [22], with StO_2_ denoting the fraction of this that is oxygenated (HbO_2_) and the remainder is deoxygenated (Hb), and ranges between 0% and 100% [16]. The *f*_blood_ is the volume fraction of tissue occupied by blood which has absorption coefficient *μ*_*a*,blood_^(*λ*)^ and is examined in the range of 0.2%–7% [16], with the remainder of tissue having background absorption *μ*_*a*,back_(*λ*) [16]. Finally, $\varepsilon_{{\text{HbO}}_{2}}(\lambda )$ and ε_Hb_(*λ*) denotes the wavelength-dependent extinction coefficients of the chromophores HbO_2_ and Hb which are found in the literature [22].

#### 2.1.2 Modified Beer–Lambert

The modified Beer–Lambert utilizes these inputs to model absorption (*A*(*λ*)) and diffuse reflectance (*R*(*λ*)) as follows:

(4) $$\begin{array}{c} A(\lambda )=L\mu_{a}(\lambda )+\mu_{s}^{\prime }(\lambda )\\ R(\lambda )=\exp(-\frac{A(\lambda )}{100}) \end{array}$$

Conventionally, *μ*_*a*_(*λ*) and $\mu_{s}^{\prime }(\lambda )$ are quoted in units of cm^−1^, however in this model units of mm^−1^ are required for diffuse reflectance units of % hence a factor of 100 is included. *L* describes a differential path length to account for the variety of photon path lengths through scattering media. *L* is often simplified to be equal to 1 [14] and $\mu_{s}^{\prime }(\lambda )$ is often modelled as a wavelength-independent constant [14, 23]. To allow for further flexibility in this model, we introduce linear scaling hyperparameters (*M*_1−3_), as shown in Equation (5), that can be fitted to MC simulations at the refractive index of interest.

(5) $$\begin{array}{l} A(\lambda )=M_{1}\mu_{a}(\lambda )+M_{2}\mu_{s}^{\prime }(\lambda )+M_{3}\\ R(\lambda )=\exp(-\frac{A(\lambda )}{100}) \end{array}$$

#### 2.1.3 Jacques 1999

The Jacques model is also based on the Beer–Lambert model, where an assumption is made that the ensemble of path lengths experienced by photons in a tissue can be approximated by a single wavelength-dependent path length *L*(*λ*) = *A*(*λ*) and (*λ*) where *A*(*λ*) and d(*λ*) are defined in Equation (6). This results in the following model with the hyperparameters (*M*_1–3_) which the authors fit to Adding Doubling simulations [15]. In our work, we refit these to MC simulations since these are considered the gold standard optical simulation method and improve the model fitting results.

(6) $$\begin{array}{l} N^{\prime }(\lambda )=\frac{\mu_{s}^{\prime }(\lambda )}{\mu_{a}(\lambda )}\\ \delta (\lambda )=\frac{1}{\sqrt{3\mu_{a}(\lambda )[\mu_{a}(\lambda )+\mu_{s}^{\prime }(\lambda )]}}\\ A(\lambda )=M_{1}+M_{2}\exp[\frac{\ln(N^{\prime }(\lambda ))}{M_{3}}]\\ R(\lambda )=\exp[-A(\lambda )\delta (\lambda )\mu_{a}(\lambda )] \end{array}$$

#### 2.1.4 Yudovsky 2009

The original Yudovsky model presents a complex analytical derivation [16] with a simplified formulation subsequently presented in their Erratum [24]. The model takes the reduced albedo (*w*^'^(*λ*)) as input and returns the diffuse reflectance spectra (*R*(*λ*)). This provides an easily applicable model with hyperparameters (*M*_1-6_) which are quoted for a refractive index (*n*) of 1.44. We found that these can be fitted to MC spectra to allow for use with other refractive indices.

(7) $$\begin{array}{l} w^{\prime }(\lambda )=\frac{\mu_{s}^{\prime }(\lambda )}{\mu_{a}(\lambda )+\mu_{s}^{\prime }(\lambda )}\\ R=M_{1}+M_{2}\exp[M_{3}w^{\prime }{\left(\lambda \right)}^{M_{4}}]+\frac{M_{5}}{1.02-M_{6}} \end{array}$$

#### 2.1.5 Fitting model hyperparameters

All analytical models considered use hyperparameters to account for the media’s refractive index. A MC dataset is generated for each refractive index as in Section 2.2.1. To fit the hyperparameters, the ground truth tissue parameters are inputted into an analytical model for each spectrum in the dataset and a single set of hyperparameters are fitted using a non-linear least squares fitting approach.

### 2.2 Generating reference datasets of diffuse reflectance spectra

In this work, we aim to compare the three models discussed in Section 2.1 against datasets with known ground truth. These datasets of diffuse reflectance spectra are generated either by simulation using MC (Section 2.2.1) or measurement of controlled, gelatin-based, tissue phantoms (Section 2.2.2).

#### 2.2.1 Monte Carlo simulations

The models take *μ*_*a*_(*λ*) and $\mu_{s}^{\prime }(\lambda )$ as inputs. These are given for bulk tissue in Equations (3) and (2) and used to generate MC simulated reference spectra. MC takes *μ*_*s*_(*λ*) as input, not $\mu_{s}^{\prime }(\lambda )$, so a random value of anisotropy (*g*) is chosen per spectrum between 0.7 and 0.9 [16] and used in Equation (1) for conversion to *μ*_*s*_(*λ*). The variable parameters are *a, b*, StO_2_, and *f*_blood_ which are bounded to 8–70 cm^−1^, 0.1–3.3, 0%–100%, 0.2%–7%, respectively, to mimic biological tissue [16, 20]; a value of each of these variables is randomly selected within these bounds to generate each of the 100 simulated spectra, meaning 100 values of each parameter are sampled. When fitting the inverse models to these spectra for parameter extraction (as described in Section 2.3), the same bounds are imposed on the fitting routine. These simulated spectra are generated using CUDAMCML [25], which is a GPU accelerated adaptation of the well-established MCML program [26]. It has been shown that the semi-infinite approximation is valid for thicknesses of above 1 cm [27] so 100 spectra were simulated for each refractive index of 1.33, 1.35, and 1.44, by propagating 100 000 photons through the semi-infinite slabs approximated by a thickness of 3 cm. The refractive indices were chosen to represent the common phantom and tissue use-cases of these models: 1.33 for use with water-based phantoms, 1.35 for use with gelatin-based phantoms (as in this work), and 1.44 for use with biological tissue.

#### 2.2.2 Controlled gelatin-based tissue phantoms

Here we construct controlled, optical tissue phantoms with well-characterized components. By measuring these phantoms we constructed a dataset of measured spectra with well-defined ground truth which can be modelled using the analytical models.

##### Phantom composition and synthesis

**Figure 1:**
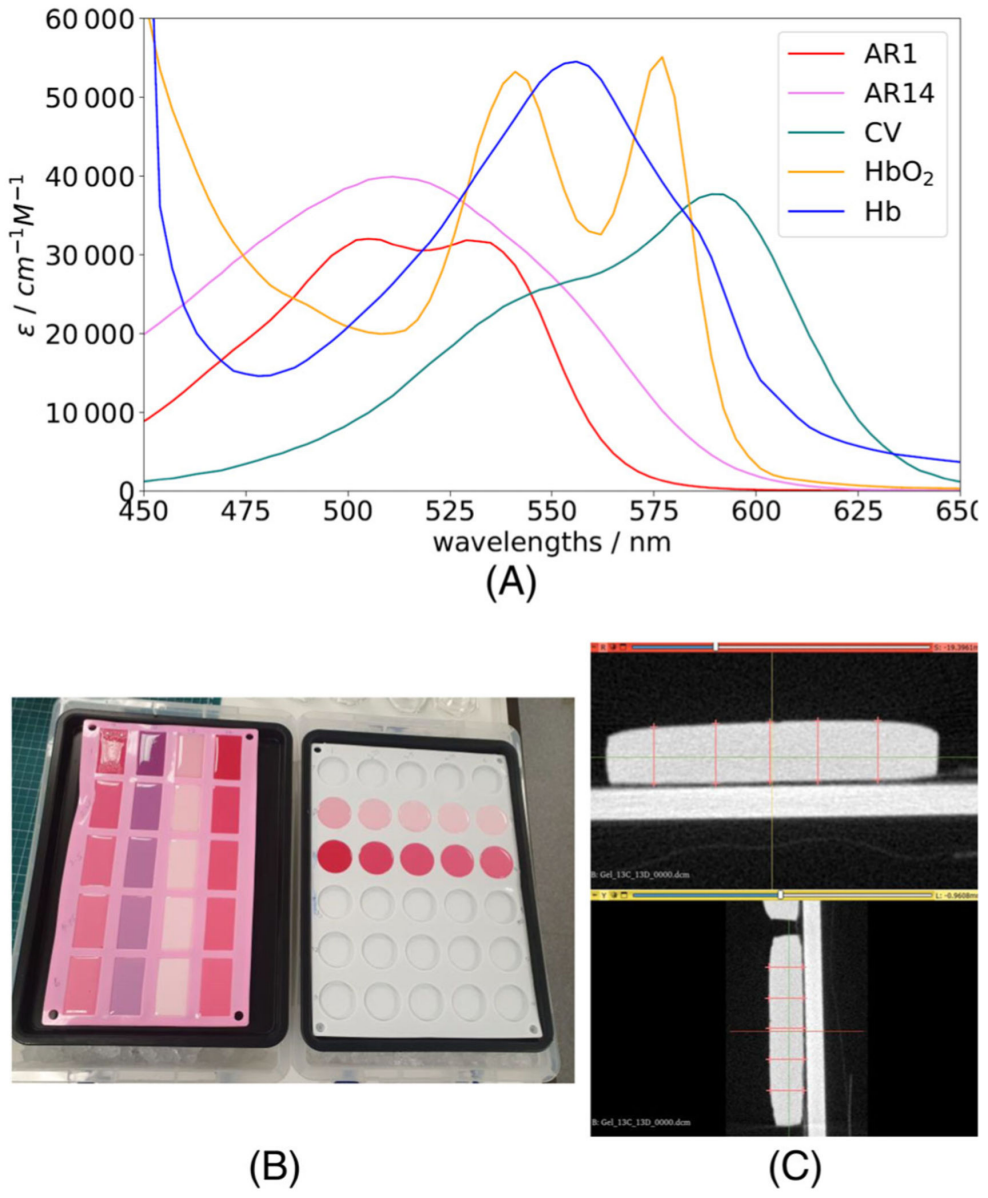
Figure regarding phantom development. (A) Effective extinction coefficients of acid red 1 (ARI), acid red 14 (AR14), and crystal violet (CV) dyes (used to synthesis gelatin-based phantoms) are displayed with the extinction coefficients of oxygenated (HbO_2_) and deoxygenated hemoglobin (Hb) (key tissue chromophores). (B) Image of 1 cm depth (left) and 5 mm depth (right) phantoms setting in ice bath. (C) An example of a CT scan acquired for the purpose of measuring thickness of a tissue phantom is shown with 10 digital measurements (mean = 6.11 mm).

The dyes acid red 1 (AR1, 210 633, Merck, Germany) and acid red 14 (AR14, B22328, Fisher Scientific, England) are chosen to mimic the extinction coefficients of oxygenated and deoxygenated hemoglobin, respectively, with a third dye of crystal violet (CV, C6158, Merck, Germany) chosen to investigate the effect of including further chromophores. As in the modelled tissue *μ*_*a*_(*λ*) (Equation (3)), the total dye concentration is modelled independently of the relative ratios of each dye. To ensure the absorbance impact of each dye is approximately equal, a factor of $\frac{5}{3}$ is included for AR14 and $\frac{1}{2}$ for CV computationally by combining with the extinction coefficients to create effective extinction coefficients (ε_eff_) that have approximately equal impact. To reflect this experimentally, the concentrations of these dyes are modified by these factors. These effective extinction coefficients calculated from literature values [28], alongside those of hemoglobin [29], can be seen in Figure 1A.

The tissue phantoms are constructed with three overall dye concentrations corresponding to fractions of blood of 0.344%, 3.44%, and 6.88%, that is, 8 × 10^−6^ mold m^−3^, 8 × 10^−5^ mold m^−3^, and 1:6 × 10^−4^ mold m^−3^. This can be described as a X1, X10, and X20 concentration with a scale factor of 8 × 10^−6^. Two-dye configurations are constructed to investigate a range of AR1:AR14 ratios with some 3-dye configurations added.

Intralipid is used to modulate the scattering coefficient of these phantoms which can be modelled with a Mie scattering function (Equation (2)) using parameters within the range of tissues. Five volume fractions of intralipid are chosen between 1% and 6% to cover a range of scattering parameters within those seen in biological tissue [20].

Each phantom consists of 6% gelatin (Type A approximately 175 g bloom, G2625, Merck, Germany) by mass and 0.5% of 4% formaldehyde (J60401, Fisher Scientific, England) to increase the melting point of the phantoms and allow their use at room temperature [18]. The remainder of each phantom consists of the dye solutions in a variety of ratios combined with intralipid at a variety of concentrations.

Phantoms are constructed with the ratios given in Table 1, where each configuration is constructed with each intralipid concentration.

For each dye, stock solutions are made at double the required concentrations: ×2, ×20, and ×40 in phosphate buffered saline (PBS, P4417, Merck, Germany) to allow for 1:1 dilution with intralipid as the final step. The stock concentrations are multiplied by a factor of $\frac{5}{3}$ for Acid Red 14 and $\frac{1}{2}$ for Crystal Violet to ensure similar absorbance impact. For each intralipid concentration, the stock solutions are made at double the required volume fractions: 2%–12% to allow for 1:1 dilution with the dye solutions as the final step. The dye solutions combined in the correct ratios are heated to 45°C–50°C with 12% gelatin (i.e., double the required amount) until solvation. The solution is cooled to below 40°C where 1% formaldehyde (i.e., double the quantity) is added and the solution is combined in equal quantities with the solution with double the desired intralipid concentration. This final solution now has the intended concentration of all constituents and is poured into a silicon mold in an ice bath, as seen in Figure 1B, to ensure homogeneity in the final phantom by rapidly setting prior to any density separation. Once cooled to below 10°C these are placed in a fridge at 4°C to fully set for at least 7 days before measurement.

Each dye solution is also combined with gelatin with a 0% intralipid solution for absorbance measurements. Additionally, each intralipid solution is combined with a gelatin solution with no dyes to allow analysis of scattering due to intralipid concentration.

Molds with a depth of 1 cm are used to allow measurement of diffuse reflectance within a semi-infinite regime [27]. MC simulations were used to confirm that this thickness was sufficient to be within the semi-infinite regime. Some additional phantoms are constructed with a depth of 5 mm to allow for sufficient transmission for total transmittance measurements required for inverse adding doubling (IAD) analysis [17].

##### Spectral measurements

**Figure 2:**
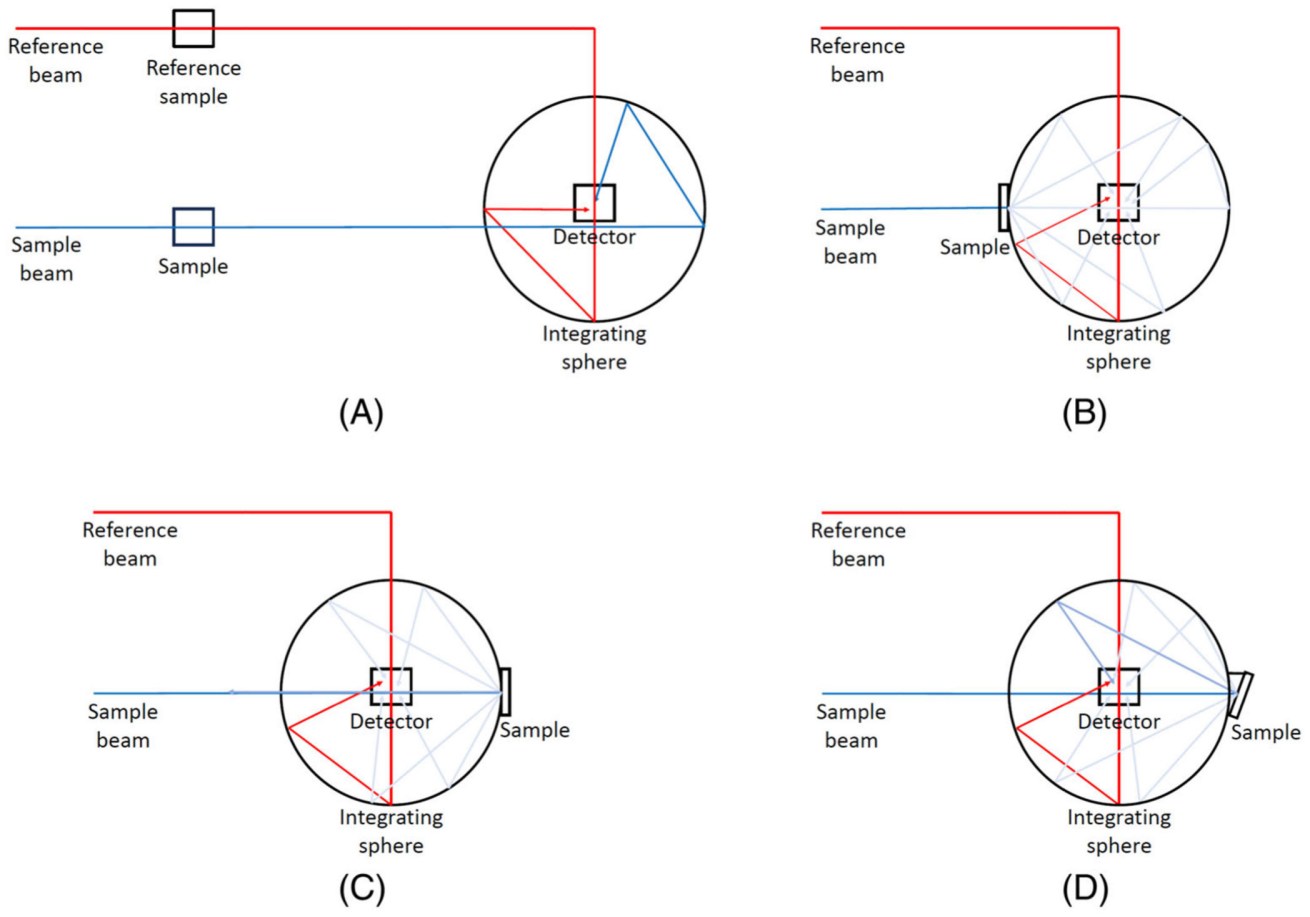
Depictions of measurement set-up for spectrophotometer measurements of gelatin based tissue phantoms of absorbance (A), total transmittance (B), diffuse reflectance (C), and total reflectance (D) where an 8° wedge is used to ensure the specular reflectance is also included.

Measurements of these phantoms are taken using a PerkinElmer Lambda 750s spectrophotometer. This dual-beam integrating sphere spectrophotometer allows for precise and accurate measurements of diffuse reflectance, total reflectance, total transmittance, and absorbance. Absorbance for each dye solution and pure dye gelatin phantom is measured with either PBS or pure gelatin as a reference, respectively. The pure gelatin absorbance is also measured. All 1 cm phantoms are used for diffuse reflectance measurements. A subset of these is also made into 5 mm phantoms alongside pure intralipid gelatin 5 mm phantoms, which are measured for total reflectance and total transmittance. All configurations are depicted for our system in Figure 2.

##### Inverse adding doubling (IAD)

IAD is used to obtain *μ*_*a*_(*λ*) and $\mu_{s}^{\prime }(\lambda )$ from total reflectance and total transmittance measurements [17]. We use this with the dual beam spectrophotometer setting and an incidence angle of 8° as per the experimental set-up in Figure 2D, with *g* fixed to 0.8 for all calculations since it was found not to change the results. Since the outputted optical properties can contain a significant number of wavelengths without convergence, a Mie scattering curve is fitted to the output $\mu_{s}^{\prime }(\lambda )$ and a second stage of IAD is run with the $\mu_{s}^{\prime }(\lambda )$ fixed to this spectrum. This returns outputs which are very similar but removing noise or errors. Further details on this two-stage IAD fitting approach can be found in previous work [30].

To obtain accurate optical properties from IAD a highly accurate sample depth must be provided. The standard measurement technique using dial calipers is not suitable in this case due to the highly compressible nature of the gelatin-based phantoms. A non-contact measurement method is therefore chosen. A CT scan is taken of each phantom using a Small Animal Radiotherapy System (SmART+, precision x-ray). A mean of 10 digital measurements is used for each phantom and an example of these measurements is shown in Figure 1C.

Finally, a commercial calibrated phantom (BioPixS) with validated ground truth optical properties at three wavelengths is used to confirm that our output *μ*_*a*_(*λ*) and $\mu_{s}^{\prime }(\lambda )$ are correct. We highlight that only the single-stage IAD algorithm can be used here as a Mie scattering curve does not accurately represent the scattering of this sample. The BioPixS phantom is measured on 3 days to demonstrate the accuracy and reproducibility of IAD-outputted optical parameters.

##### Modelling tissue phantom optical properties

The *μ*_*a*_(*λ*) of the two-dye configuration phantoms can be modeled as in Equation (8) where the background spectrum is determined by measuring the absorption of pure gelatin solution, whereas the scattering is modelled based on the intralipid concentration according to trends determined using IAD.

(8) $$\begin{array}{l} \mu_{a}(\lambda )=8\times 10^{-6}c_{\text{tot}}\ln(10)[\text{AR}1\varepsilon_{\text{AR1,eff}}(\lambda )+(1-\text{AR}1)\varepsilon_{\text{AR14,eff}}(\lambda )]\\ \;+\mu_{a,\text{background}}(\lambda ). \end{array}$$

In this equation *c*_tot_ is the total concentration of dye independent of dye identity, AR1 and AR14(= 1 − AR1) are the relative ratios of each dye, ε_AR1,eff_ (*λ*) and ε_AR14,eff_ (*λ*) are the effective extinction coefficients of each dye, and *μ*_*a*,background_(*λ*) is the measured background absorbance from gelatin. This can be adapted to a threedye configuration as follows:

(9) $$\begin{array}{l} \mu_{a}(\lambda )=8\times 10^{-6}c_{\text{tot}}\ln(10)[\text{AR}1\varepsilon_{\text{AR1,eff}}(\lambda )\\ \;+\text{AR}14\varepsilon_{\text{AR14,ef}f}(\lambda )+(1-\text{AR}1-\text{AR}14)\varepsilon_{\text{CV,eff}}(\lambda )]\\ \;+\mu_{a,\text{background}}(\lambda ), \end{array}$$

where CV(= 1 − AR1 − AR14) is the relative ratio of crystal violet and ε_CV,eff_ (*λ*) is the effective extinction coefficient of this dye.

### 2.3 Evaluation of model performance

In this work, the above models are evaluated against spectra with known ground truth. These reference spectra are either found by MC simulation or measurement of controlled phantoms, however the evaluation of each is broadly the same. The forward models are evaluated in terms of the fit of their predicted spectra, and the inverse problem solutions (parametric fits) are investigated in terms of the quality of parameter extraction.

Diffuse reflectance spectra can be predicted by inputting ground truth values into each forward tissue model and compared with corresponding reference (MC/measured) spectra. The Normalized Root Mean Squared Error (NRMSE), defined in Equation (10), is calculated to quantitatively evaluate the similarity between the reflectance predicted by the forward model and the associated reference (MC/measured) reflectances. In the context of this work the wavelength ranges considered for this metric correspond to those ranges used for fitting the inverse models.

(10) $$\text{NRMSE}=\frac{\sqrt{\frac{1}{\Lambda }\sum_{\lambda }^{\Lambda }{\left(s_{\lambda }-r_{\lambda }\right)}^{2}}}{\sqrt{\frac{1}{\Lambda }\sum_{\lambda }^{\Lambda }r_{\lambda }^{2}}}.$$

Here the modelled spectrum *s*_*λ*_ at any given wavelength *λ* is evaluated against the intensity at the same wavelength in the reference (MC/measured) spectrum *r*_*λ*_ using the normalized root mean squared error (NRMSE) calculated across all Λ wavelengths. These forward model reflectance spectra are also plotted against the reference (MC/measured) reflectance spectra and a regression line calculated. This is evaluated using the Pearson correlation coefficient (*r*) and the *p*-value (*p*) for a hypothesis test with the null hypothesis that there is no correlation. Using a 95% confidence interval, significant *p* is <0.05, whereas a strong correlation is demonstrated by *r* close to 1. This gives an indication of similarity in shape, while disregarding offsets.

The inverse problem solutions can also be evaluated by determining how well they recover the ground truth input parameters using a non-linear least squares fitting approach (using SciPy v1.10.0 scipy.optimize. least_squares function). The cost function for this is shown in Equation (11) where *R*(*λ*) is the reference (MC/measured) spectrum and *M* is the forward model function. This equation is modified to fit the parameters used to calculate *μ*_*a*_(*λ*) and $\mu_{s^{\prime }}(\lambda )$ directly.

(11) $${\text{argmin}}_{\mu_{a}(\lambda ),\mu_{s}^{\prime }(\lambda )}\sum {(R-M\left(\mu_{a}(\lambda ),\mu_{s}^{\prime }(\lambda )\right))}^{2}.$$

All models are fitted by only considering wavelengths up to 600 nm as this is the region the Yudovsky model has been shown to be effective by the authors [31]. When fitting to measure phantom spectra, only wavelengths up to 575 nm are considered due to the forward model predicted spectra being highly unreliable after this point for all three models as discussed in Section 3.2. The quality of parameter recovery is examined by calculating the correlation between the fitted and ground truth parameters (using SciPy v1.10.0 scipy.stats.linregress function). This is evaluated with the Pearson correlation coefficient (*r*) and the *p*-value (*p*) as above. The quality of parameter recovery is also evaluated using absolute percentage errors (APE) as given by Equation (12) where *e* is the extracted parameter and *g* is the ground truth parameter. The median and interquartile range of these parameters are presented for each dataset.

(12) $$\text{APE}=|\frac{e-g}{g}|\times 100.$$

These evaluation methods can be done for quantitative or relative spectra. Here relative spectra are defined as mean normalized spectra. This allows parameters to be extracted considering the shape of the spectrum but without considering absolute intensity. This is investigated as it is simpler to capture relative data in clinical environments [32].

## 3 Results

### 3.1 Monte Carlo

Each model has hyperparameters which are fitted to MC datasets for refractive indices 1.33, 1.35, and 1.44. These are listed in Table 2 alongside any literature hyperparameters [15, 16]. It should be noted that the literature hyperparameters for Jacques could not be directly replicated by fitting to our MC simulations. Refitting these hyperparameters leads to the mean (±SD) NRMSE of the *n* = 1:33 dataset improving from 0.080(±0.056) to 0.021(±0.028). In contrast, Yudovsky’s literature hyperparameters are similar to our refitting. For Yudovsky, despite the expected equivalence, we observed a small discrepancy in fitting quality between the “extensive model” [16] and the “simplified model” described in their Erratum [24]. This can be seen for a refractive index of 1.44 in Figure A1, which matches the results quoted in their Erratum [24]. The “simplified model” improves the mean (±SD) NRMSE of the *n* = 1:44 dataset from 0.050(±0.014) to 0.010(±0.003). For this reason the Erratum model is used for this work.

**Figure 3:**
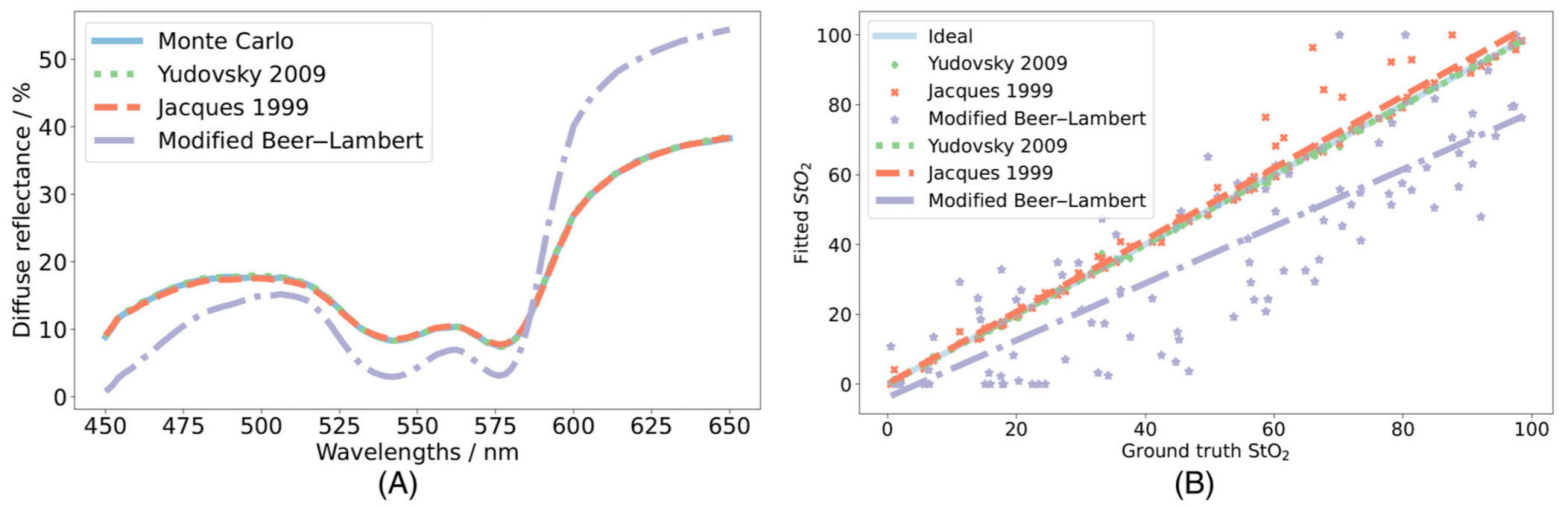
Figure depicting evaluation of forward models (A) and inverse problem solutions (B). Figure a depicts an example of the predicted spectra from each forward analytical model: Yudovsky 2009 (green dotted), Jacques 1999 (orange dashed), and Modified Beer-Lambert (purple dot-dashed), using ground truth variables for a refractive index of 1.44 compared with that predicted by MC (blue solid). Figure b shows an example of difference in quality of parameter recovery by fitting each inverse analytical model to MC simulations in the wavelength range of 450−600 nm: Yudovsky 2009 (green +), Jacques 1999 (orange ×), and Modified Beer−Lambert (purple *), and their associated trend lines for a refractive index of 1.44 for StO_2_.

Example data comparing the analytical models to MC simulations can be seen in Figure 3. For each model, spectra are generated with each input parameter set from the MC dataset and NRMSE is calculated per spectrum. The mean (±SD) of these NRMSE per model per refractive index are shown in Table 3. Only wavelengths until 600 nm are considered for these metrics as in Section 2.3. An example spectrum with each of the forward models and modelled spectra using ground truth parameters are shown in Figure 3A for a refractive index of 1.44. A regression line calculated between each forward model dataset and the MC simulated dataset for each refractive index and the correlation coefficient (*r*) can be seen in Table 3.

Finally, the inverse problems with our fixed hyperparameters are fitted as in 2.3 to this same MC dataset to recover the StO_2_, *f*_blood_, *a*, and *b* tissue parameters. A linear regression is fitted between these retrieved values and the ground truth counterparts. The Pearson *r* values of this, and the median (inter-quartile range) absolute percentage errors between the fitted and ground truth tissue parameters are shown in Table 4 for a refractive index of 1.44 with further parameters and refractive indices shown in Table A1. An example of the fitted parameters compared with the ground truth parameters is shown for StO_2_ at a refractive index of 1.44 in Figure 3B.

### 3.2 Gelatin-based tissue phantoms

**Figure 4:**
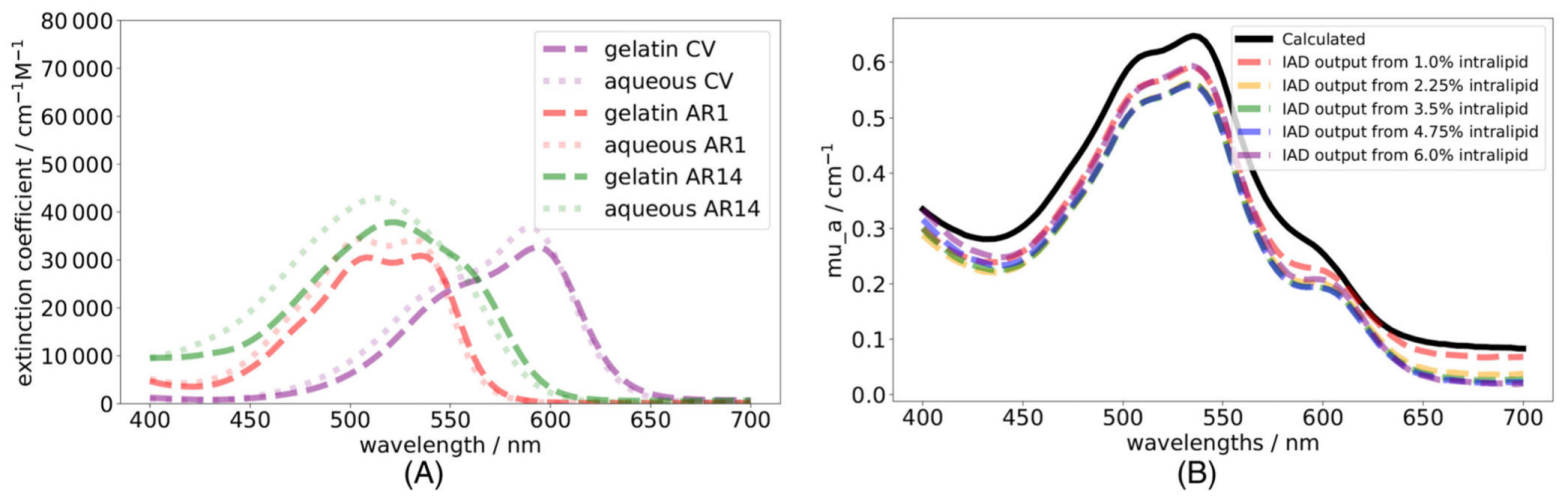
Figure a shows ε_eff_ (*λ*) calculated for each dye [acid red 1 (AR1), acid red 14 (AR14), crystal violet (CV)] measured in gelatin (dashed) or PBS (dotted) demonstrating the shift in peaks. Figure b shows a calculated (black solid) *μ*_*a*_(*λ*) for one 3-dye configuration compared with the IAD output *μ*_*a*_(*λ*) (colored dashed) from measurements of phantoms in this configuration with a range of intralipid concentrations.

Each dye absorbance is measured in both an aqueous and gelatin based solution with effective extinction coefficients calculated in each case. Whilst the aqueous measurements closely match the expected ε_eff_ (*λ*) from literature [28], the gelatin measurements show a shifting of peaks, as seen in Figure 4A. This is likely due to interaction of the dyes with gelatin altering their interactions with solvent as has been noted with other dyes [33]. Since these peak shifts are seen in all subsequent data, these gelatin-based ε_eff_ (*λ*) are used in the model analysis. A pure gelatin solution absorption is measured at the concentration used in each phantom. This is used as a background *μ*_*a*_(*λ*), as seen in Figure A2, to account for any absorption not due to dyes. We further assume that intralipid is a purely scattering medium. This is considered a reasonable assumption since the IAD returned *μ*_*a*_ for the purely intralipid phantoms are similar to the pure gelatin background *μ*_*a*_ seen in Figure A2.

IAD [17] is used to analyze phantoms of 5 mm depth with or without dyes in all intralipid concentrations. The *a* and *b* Mie coefficients fitted to the scattering in these cases are plotted against intralipid concentration. It was found that *b* had no significant trend with intralipid concentration and so a median value of 0.98 was used in this work. However, a clear trend was identified for *a* as can be seen in Figure A3. This trend is described in Equation (13), where *I* is the intralipid concentration and *I*_0_ is the reference concentration of 1%v/v:

(13) $$a=6.66cm^{-1}\frac{I}{I_{0}}+2.55cm^{-1}.$$

A Pearson correlation coefficient (*r*) of 0.99 and *p* of 0.00 was observed, therefore this is used throughout this work to model scattering based on intralipid concentration.

The calculated *μ*_*a*_(*λ*) using ε_eff_ (*λ*) displayed in Figure 4A is compared with the IAD outputs of phantoms including dyes with each intralipid concentration. An example of this is seen in Figure 4B. This shows the overall spectrum is accurate, however the peak for CV (~600 nm) appears shifted in wavelength, likely due to interactions with intralipid which cannot be captured in absorbance measurements.

**Figure 5:**
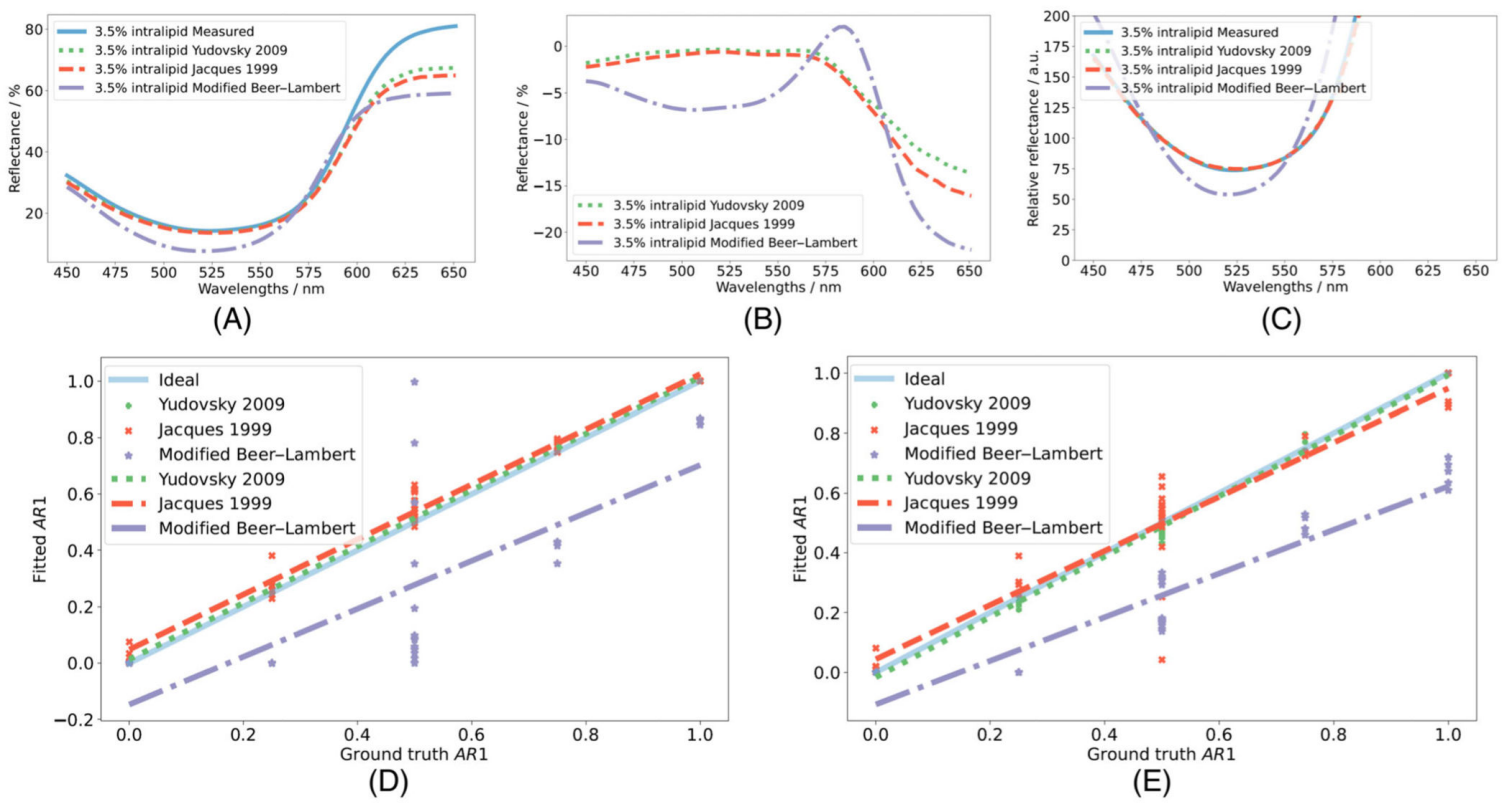
Example of measured (blue) spectrum for one 2-dye configuration at an intralipid concentration of 3.5% compared with the respective spectra generated from the Yudovsky 2009 (green dotted), Jacques 1999 (orange dashed), and Modified Beer–Lambert (purple dot-dashed) models using the ground truth parameters for quantitative (A) or relative (C) spectra (mean normalized in the range 450–575 nm). The residuals between the modelled quantitative spectra and the measured spectra are shown in B. Examples of the parameter recovery from the measured phantom spectra for the AR1 proportion by Yudovsky 2009 (green +), Jacques 1999 (orange ×), and Modified Beer–Lambert (purple) models compared with the ground truth parameters (blue solid) for fits to quantitative (D) or relative (E) spectra in the wavelength range 450–575 nm.

Using a refractive index of 1.35 [18] diffuse reflectance spectra are generated from each model using *μ*_*a*_(*λ*) and $\mu_{s^{\prime }}(\lambda )$ from Equations (8) and (2) using the trend in Equation (13) and the median *b* value for each 2-dye configuration and intralipid concentration. The forward Yudovsky, Jacques, and Modified Beer–Lambert models in Section 3.1 are compared with diffuse reflectance measurements for each phantom. An example of this can be seen in Figure 5A for quantitative spectra or Figure 5C for mean normalized relative data. The data is normalized using the mean of the wavelength range 450–575 nm as beyond this region the models appear to fit poorly as seen in Figure 5B. The mean NRMSE (±SD) between each spectrum and the measured spectrum, in the region 450–575 nm, for this dataset can be seen in Table 5. The Pearson correlation coefficient *r* between the forwards spectra compared with the measured spectra for each model is shown in Table 5 and further parameters. Whilst the overall magnitude of these is higher, the trend in quality of fit is similar as to the MC simulations.

The inverse problems are solved by non-linear least squares approaches using measured spectrum as reference observations. The recovered parameters are correlated to the ground truth parameters. The associated errors and correlation coefficients can be seen in Table 6 (and further in Table A2) and an example of the fitted parameters compared with ground truth parameters is shown for AR1 in Figure 5D for fits to quantitative spectra or Figure 5E for fits to relative spectra.

Finally, some 3-dye configurations are investigated with the addition of CV. Some examples of both quantitative and relative spectral reconstruction using the ground truth parameters in the forward Yudovsky model for a single 3-dye configuration at a range of intralipid concentrations can be seen in Figure A4. Despite considering the shift in CV peak due to gelatin, there appears to be a further shift, likely due to interactions with intralipid. This can be seen as an offset between the measured and reconstructed spectra in Figure A4 and appears to change with intralipid concentration. Due to the limited ground truth parameter range for these 3-dye configurations, only median percentage errors are presented for the inverse problem solution performance in Table A3 alongside their interquartile range. These show a dramatic loss in accuracy of AR1 and AR14 recovery, despite good CV recovery, likely due to the imperfect CV absorbance modelling distorting the spectrum.

## 4 Discussion and Future Work

When compared with MC simulations, the semiempirical Yudovsky model performs best in terms of fit and parameter extraction, followed closely by the Jacques model. The Modified Beer–Lambert model, however, performs much more poorly. It is also noted that the Jacques model fits MC simulations significantly better after refitting the hyperparameters, however the Yudovsky model performs well using literature values provided that the simplified erratum model is used.

When modelling tissue phantom spectra, it is important to have some prior knowledge of the phantoms. This allows accurate *μ*_*a*_(*λ*) and $\mu_{s^{\prime }}(\lambda )$ calculation, for example, utilizing the shift in dye peaks from their literature data, background gelatin absorbance, and the trend in Mie scattering parameters with intralipid concentration. Some variation is seen in IAD which may be due to inaccuracies in sample depth measurement or variations in spectral measurements. This suggests that it gives an indication of the optical properties of a sample, however, it may not be precise.

When modelling the measured tissue phantom diffuse reflectance spectra using the forward models, Yudovsky performs best followed closely by Jacques, with Modified Beer–Lambert performing significantly worse. The NRMSE calculated for relative data is an improvement on those calculated for quantitative data, suggesting that these models are reproducing the shape of the spectra well even when relatively constant offsets are present. When introducing a third dye (CV) the absorbance peak of this dye is noticeably wavelength shifted in experimental measurements compared with the theoretical spectra which then appears distorted. This shift appears to change with varying concentrations of intralipid suggesting some interaction there.

When fitting the models to experimental data for parameter recovery, Yudovsky and Jacques produce excellent parameter recovery for AR1 and AR14 in both quantitative and relative regimes. For *c*_tot_ and *I* no models were able to recover the parameters well, whereas all parameters were recovered well by Yudovsky and Jacques when fitted to MC data. This is suggestive of some overlap in the effects of *c*_tot_ and *I* on the spectra. In the three-dye configuration CV is recovered reasonably, despite the peak being outside of the region considered for fitting. The shift in the CV peak, however, distorts the rest of the spectrum leading to very poor recovery of all other parameters. This demonstrates the importance of prior understanding of the chromophores for any model to recover parameters accurately.

This work is associated with some limitations. First, while every effort was made to synthesize optical phantoms resembling biological tissue, measurements of true biological tissue could not be used for this work due to a lack of reliable ground truth parameters in tissue. Similarly, hemoglobin chromophores could not be utilized in the phantoms due to their oxygen sensitivity which could not be controlled within the spectrophotometer measurement set-up. This led us to rely on stable synthetic dyes instead, which do not perfectly replicate the spectra of oxy and deoxyhemoglobin. The major dye peaks maxima are ~45 nm lower than the distinctive hemoglobin peaks which may contribute to different wavelength regions performing differently with these phantoms compared with biological samples. The factors chosen to allow approximately equal impact from each dye were chosen to ease synthesis and should be optimized. Since these factors are incorporated in both synthesis and analysis, it is believed that these do not impact the conclusions of this study. The difference in extinction coefficient found when measuring each dye in gelatin compared with aqueous solution is not fully understood and requires further study. These phantoms are also spatially uniform meaning that, whilst these could be used to determine the performance of these models spatially using hyperspectral imaging systems, other methods would be required to assess spatial resolution of these systems [34, 35]. Second, the shift in absorbance of the third chromophore made it difficult to model thereby limiting the investigation of the impact of additional chromophores. Third, while the IAD method produces results similar to our ground truth, there remain some differences between IAD output and the expected ground truth. This could impact the quality of the intralipid scattering model used in this work. Fourth, these models assume a planar surface for measurement, however, the surface may deviate from this. In these cases, spatial frequency domain methods may be an alternative to obtain accurate *μ*_*a*_ and $\mu_{s^{\prime }}$ values for tissues [36]. Finally, the precise measurement of extinction coefficients of hemoglobin in biological tissue is not possible due to the scattering nature of these media. However, it is shown in our work that the medium chosen for the dye can have an impact on the extinction coefficients and therefore the quality of the models. For this reason, the optical properties of tissue must be well defined to mimic the quality of results in this work.

Future work should include investigation of these models for use with hyperspectral imaging cameras which may not have as densely sampled spectra as these have been shown to be likely methods of obtaining these diffuse reflectance spectra intra-operatively [37]. The impact of the medium on the dye extinction coefficients should be further investigated and understood. There could also be greater investigation of including further chromophores whose absorbance behavior is well modelled and optimization of the fitting region. Two-layer models should also be considered for tissue structures which exist in layered configurations, such as skin. Finally, similar analyses could be conducted in other wavelength ranges including the near infra-red range which can also be used for perfusion analysis with bespoke light sources [38].

In conclusion, the Yudovsky single layer model works well for modelling of tissue that can be approximated by a semi-infinite, homogeneous slab. Jacques is also able to well approximate this with a simpler model. All models require appropriate prior knowledge of the *mu*_*a*_ and $mu_{s}^{\prime }$ properties of the tissues to allow for a good quality of parameter recovery.

## Supplementary Material

Additional supporting information can be found online in the Supporting Information section at the end of this article.

Supplementary material

## Figures and Tables

**Table 1:** Table displaying the percentage of each dye [acid red 1 (AR1), acid red 14 (AR14), crystal violet (CV)] a used for each dye configuration alongside the total dye scaled concentration in arbitrary units.

Total dye scaled Concentration (arbitrary units)	Dye percentage (%)		
	AR1	AR14	CV
1	50	50	0
1	50	25	25
1	25	25	50
10	100	0	0
10	75	25	0
10	50	50	0
10	25	75	0
10	0	100	0
10	25	50	25
10	50	25	25
10	25	25	50
20	50	50	0
20	50	25	25
20	25	25	50

**Table 2:** Table displaying single layer model hyperparameters fitted to MC datasets of each refractive index (black) with available literature values (gray shaded).

Model	Parameter	Refractive index				
		1.33		1.35	1.44	
Yudovsky 2009	*M*_1_	−0.0253		−0.0257	−0.0254	−0.0247
	*M*_2_	0.0166		0.0159	0.0135	0.0137
	*M*_3_	2.873		2.873	2.873	2.873
	*M*_4_	1.64		1.64	1.64	1.64
	*M*_5_	0.0123		0.0124	0.0120	0.0116
	*M*_6_	1.02		1.02	1.02	1.02
Jacques 1999	*M*_1_	7.0188	6.3744	7.1185	7.0438	
	*M*_2_	0.2464	0.35688	0.2750	0.6902	
	*M*_3_	4.2241	3.4739	4.2571	4.1449	
Modified Beer-Lambert	*M*_1_	0.283		0.308	0.256	
	*M*_2_	0.009		0.008	0.014	
	*M*_3_	0.203		0.311	0.274	

**Table 3:** Mean (±SD) NRMSE (3, d.p.) between each forwards spectrum from each model and each of 100 MC simulated spectra using the same ground truth variable parameters for each refractive index dataset and each analytical model.

Model	Refractive index	NRMSE	*r*
Yudovsky 2009	1.33	0.013 (±0.006)	**1.000**
	1.35	0.013 (±0.005)	**1.000**
	1.44	0.010 (±0.004)	**1.000**
Jacques 1999	1.33	0.037 (±0.065)	**0.999**
	1.35	0.045 (±0.074)	**0.999**
	1.44	0.030 (±0.048)	**1.000**
Modified Beer-Lambert	1.33	0.630 (±0.461)	**0.667**
	1.35	0.603 (±0.339)	**0.639**
	1.44	0.675 (±0.620)	**0.605**

*Note:* This is presented with the Pearson *r* (bold if Pearson *p* < 0.05) for the linear regression between all forwards spectra against MC simulated spectra for each refractive index dataset and each analytical model. All metrics are evaluated for the wavelength region of 450–600 nm.

**Table 4:** The Pearson r (bold if p < 0.05) of the linear regression line between the fitted tissue parameters and their ground truth displayed with their median (inter-quartile range) APE.

			Median (inter-quartile range)
Parameter	Model	*r*	APE (%)
StO_2_	Y	**1.00**	0.913 (1.92)
	J	**0.986**	2.21 (4.97)
	BL	**0.838**	43.0 (49.8)
*f*_blood_	Y	**0.982**	5.68 (6.08)
	J	**0.928**	7.26 (16.2)
	BL	**0.582**	49.6 (32.2)
*a*	Y	**0.992**	3.90 (4.73)
	J	**0.959**	4.54 (15.3)
	BL	**−0.706**	40.0 (114)
*b*	Y	**1.00**	1.50 (2.92)
	J	**0.963**	2.86 (9.11)
	BL	**−0.446**	95.4 (5.68)

*Note*: This is shown for each variable and for a refractive index of 1.44 when extracted by fitting Yudovsky 2009 (Y), Jacques 1999 (J), or Modified Beer−Lambert (BL) to the Monte-Carlo dataset in the wavelength range 450−600 nm. All presented to 3 s.f.

**Table 5:** Mean (±SD) NRMSE (3. d.p.) between the modelled spectrum using the ground truth parameters and the measured spectrum of a tissue phantom for each analytical model for either quantitative or relative spectra.

Model	Quantitative (Q) or relative (R)	NRMSE	*r*
Yudovsky 2009	Q	0.059 (±0.031)	**0.998**
	R	0.012 (±0.009)	**0.999**
Jacques 1999	Q	0.075 (±0.032)	**0.998**
	R	0.022 (±0.023)	**0.995**
Modified Beer-Lambert	Q	0.464 (±0.294)	**0.808**
	R	0.215 (±0.139)	**0.930**

*Note*: Alongside this is the Pearson *r* (bold if *p* < 0.05) for each model for all forwards spectra compared with the measured spectra. All metrics evaluated for the wavelength range 450–575 nm.

**Table 6:** The Pearson *r* (bold if *p* < 0.05) of the linear regression line between the fitted parameters and their ground truth displayed with their median (inter-quartile range) absolute percentage errors for each variable when extracted by fitting Yudovsky 2009 (Y), Jacques 1999 (J), or Modified Beer–Lambert (BL) for both quantitative and relative data.

Parameter	Model	Quantitative (Q) or relative (R)	*r*	Median (inter-quartile range)
				APE (%)
*AR*1	Y	Q	**0.997**	1.59(11.0)
		R	**0.998**	4.42 (8.31)
	J	Q	**0.989**	7.02 (20.2)
		R	**0.934**	10.4 (23.7)
	BL	Q	**0.756**	83.5 (57.0)
		R	**0.939**	50.0 (31.4)
*AR*14	Y	Q	**1.00**	1.36 (8.69)
		R	**0.998**	2.48 (5.76)
	J	Q	**0.989**	7.02 (16.3)
		R	**0.934**	6.34 (10.0)
	BL	Q	**0.756**	80.4 (72.1)
		R	**0.939**	37.5 (66.7)
I	Y	Q	**0.648**	125 (276)
		R	0.310	93.9 (30.0)
	J	Q	**0.540**	122 (288)
		R	**−0.451**	100 (55.7)
	BL	Q	**−0.705**	100(172)
		R	−0.157	100 (0.00)
*c*_tot_	Y	Q	**0.500**	102 (226)
		R	0.303	66.1 (46.8)
	J	Q	**0.684**	100 (260)
		R	−0.0959	89.7 (267)
	BL	Q	**0.721**	35.2 (21.6)
		R	**0.689**	44.6 (23.6)

*Note*: All metrics calculated for the wavelength range 450–575 nm and presented to 3 s.f.

## Data Availability

The data that support the findings of this study are available from the corresponding author upon reasonable request.
