# Supplementary material for: A comparative study of analytical models of diffuse reflectance in homogeneous biological tissues: Gelatin-based phantoms and Monte Carlo experiments
Source: J Biophotonics. Author manuscript; Available in PMC 2025 Aug 16. (PMC7618019; doi:10.1002/jbio.202300536)
Supplement: Supplementary material [file EMS207646-supplement-Supplementary_material.pdf]

APPENDIX

FIGURE 1 Example of difference in forwards model similarity to Monte Carlo simulation (blue solid) between the literature extensive Yudovsky 2009 (red dashed) and simplified Yudovsky 2009 (green dotted) models using ground truth variables for a refractive index of 1.44.

TABLE 1 The gradient m, offset c, Pearson r, and p values of the linear regression line between the fitted tissue parameters and their ground truth displayed with their median (inter-quartile range) absolute percentage errors. This is shown for each variable and for each refractive index dataset when extracted by fitting Yudovsky 2009 (Y), Jacques 1999 (J), or Modified Beer-Lambert (BL) to the Monte-Carlo dataset. All presented to 3s.f.

parameter	model	refractive	m	c	r	p	median (inter-quartile range)
		index	(ideal =1)	(ideal = 0)	(ideal = 1)	(ideal = 0)	absolute percentage error (%)
StO_2	Y	1.33	1.00	-0.373	1.00	$2.39{\times}10^{-150}$	1.23 (3.01)
		1.35	0.999	0.0672	1.00	$4.93{\times}10^{-149}$	0.815 (2.10)
		1.44	0.999	-0.074	1.00	$4.87{\times}10^{-153}$	0.913 (1.92)
	J	1.33	1.00	1.733	0.979	$1.44{\times}10^{-69}$	3.40 (9.43)
		1.35	1.03	1.03	0.980	$2.18{\times}10^{-70}$	3.97 (12.9)
		1.44	1.04	0.207	0.986	$1.64{\times}10^{-77}$	2.21 (4.97)
	BL	1.33	0.712	1.56	0.855	$1.17{\times}10^{-29}$	39.4 (38.5)
		1.35	0.679	3.92	0.838	$3.87{\times}10^{-30}$	33.3 (31.4)
		1.44	0.766	-2.73	0.838	$1.70{\times}10^{-27}$	43.0 (49.8)
f_{blood}	Y	1.33	0.976	-0.0206	0.980	$2.76{\times}10^{-70}$	5.74 (6.19)

		1.35	0.962	0.0365	0.975	8.29×10^{-66}	4.58 (6.98)
		1.44	0.927	0.152	0.982	3.60×10^{-73}	5.68 (6.08)
	J	1.33	1.17	0.187	0.915	2.21×10^{-40}	9.51 (28.7)
		1.35	1.16	0.167	0.912	9.18×10^{-40}	11.0 (20.1)
		1.44	1.15	0.141	0.928	1.10×10^{-43}	7.26 (16.2)
	BL	1.33	0.487	0.431	0.641	7.16×10^{-13}	46.4 (28.2)
		1.35	0.461	0.478	0.575	3.99×10^{-10}	45.8 (41.8)
		1.44	0.339	0.652	0.582	2.05×10^{-10}	49.6 (32.2)
a	Y	1.33	0.948	0.145	0.993	2.58×10^{-93}	5.06 (5.92)
		1.35	0.968	-0.192	0.995	4.71×10^{-102}	4.05 (5.57)
		1.44	1.05	-2.59	0.992	1.67×10^{-89}	3.90 (4.73)
	J	1.33	0.909	7.23	0.952	4.13×10^{-52}	7.09 (22.0)
		1.35	0.929	5.72	0.966	3.04×10^{-59}	9.16 (16.2)
		1.44	0.934	5.88	0.959	1.05×10^{-55}	4.54 (15.3)
	BL	1.33	-0.197	74.7	-0.554	2.23×10^{-9}	63.8 (133)
		1.35	-0.142	73.1	-0.448	2.89×10^{-6}	103 (206)
		1.44	-0.488	78.1	-0.706	2.28×10^{-16}	40.0 (114)
b	Y	1.33	0.978	0.0663	0.998	3.53×10^{-121}	1.38 (2.47)
		1.35	0.989	0.0314	0.998	5.55×10^{-125}	1.82 (2.85)
		1.44	0.991	0.0185	0.999	1.33×10^{-140}	1.50 (2.92)
	J	1.33	0.977	0.216	0.910	3.76×10^{-39}	3.45 (16.0)
		1.35	1.01	0.212	0.906	2.35×10^{-38}	5.29 (25.2)

		1.44	1.01	0.120	0.963	1.15×10^{-57}	2.86 (9.11)
	BL	1.33	-0.104	0.360	-0.413	1.92×10^{-5}	95.2 (4.85)
		1.35	-0.0888	0.303	-0.394	5.02×10^{-5}	94.1 (5.60)
		1.44	-0.0859	0.327	-0.446	3.38×10^{-6}	95.4 (5.68)

FIGURE 2 Background μ_a using absorption of pure gelatin solution compared to the IAD returned μ_a of the phantoms containing intralipid but no dyes.

FIGURE 3 Trend in a with intralipid concentration alongside linear regression. The trend in b with intralipid concentration is also plotted alongside the median b value.

TABLE 2 The regression gradient m, offset c, Pearson r, and p values, and the median (inter-quartile range) absolute percentage errors for each variable when extracted by fitting Yudovsky 2009 (Y), Jacques 1999 (J), or Modified Beer-Lambert (BL) to measured tissue phantom spectra for both quantitative and relative data. All presented to 3s.f.

parameter	model	Quantitative (Q)	*m*	*c*	*r*	*p*	median (inter-quartile range)
		or Relative (R)	(ideal =1)	(ideal = 0)	(ideal = 1)	(ideal = 0)	absolute percentage error (%)
*AR*1	Y	Q	1.00	0.0132	0.997	5.75×10^{-38}	1.59 (11.0)
		R	1.01	-0.0166	0.998	6.72×10^{-40}	4.42 (8.31)
	J	Q	0.976	0.0481	0.989	6.16×10^{-29}	7.02 (20.2)
		R	0.905	0.0442	0.934	2.67×10^{-16}	10.4 (23.7)
	BL	Q	0.850	-0.148	0.756	1.50×10^{-7}	83.5 (57.0)
		R	0.730	-0.107	0.939	7.84×10^{-17}	50.0 (31.4)
*AR*14	Y	Q	1.00	-0.0140	0.997	5.75×10^{-38}	1.36 (8.69)

		R	1.01	0.00675	0.998	6.72×10^{-40}	2.48 (5.76)
	J	Q	0.976	-0.0244	0.989	6.16×10^{-29}	7.02 (16.3)
		R	0.905	0.0508	0.934	2.67×10^{-16}	6.34 (10.0)
	BL	Q	0.850	0.297	0.756	1.50×10^{-7}	80.4 (72.1)
		R	0.730	0.377	0.939	7.84×10^{-17}	37.5 (66.7)
I	Y	Q	2.41	1.09	0.648	2.61×10^{-5}	125 (276)
		R	0.998	1.37	0.310	6.96×10^{-2}	93.9 (30.0)
	J	Q	1.62	3.73	0.540	8.11×10^{-4}	122 (288)
		R	-0.767	4.76	-0.451	6.61×10^{-3}	100 (55.7)
	BL	Q	-1.75	11.8	-0.705	2.29×10^{-6}	100 (172)
		R	-0.484	3.94	-0.157	3.66×10^{-1}	100 (0.00)
c_{tot}	Y	Q	1.46	9.25	0.500	2.23×10^{-3}	102 (226)
		R	0.917	4.85	0.303	7.72×10^{-2}	66.1 (46.8)
	J	Q	2.00	6.76	0.684	5.95×10^{-6}	100 (260)
		R	-0.265	27.8	-0.0959	5.84×10^{-1}	89.7 (267)
	BL	Q	0.356	3.02	0.721	1.01×10^{-6}	35.2 (21.6)
		R	0.309	3.23	0.689	4.63×10^{-6}	44.6 (23.6)

FIGURE 4 Examples of Measured (blue) spectrum for one 3-dye configuration at a variety of intralipid concentrations compared to the respective spectra generated from the Yudovsky 2009 (green dotted) model using the ground truth parameters to generate quantitative (a) or relative (b) spectra.

TABLE 3 The median (inter-quartile range) absolute percentage errors for each variable when extracted by fitting Yudovsky 2009 (Y), Jacques 1999 (J), or Modified Beer-Lambert (BL) to measured 3-dye tissue phantom spectra for both quantitative and relative data. All presented to 3s.f.

parameter	model	Quantitative (Q)	median (inter-quartile range)
		or Relative (R)	absolute percentage error (%)
*AR*1	Y	Q	22.7 (25.8)
		R	14.9 (9.14)
	J	Q	30.6 (29.8)
		R	49.1 (84.6)
	BL	Q	86.7 (58.3)
		R	77.1 (63.5)
*AR*14	Y	Q	25.3 (23.0)
		R	40.4 (23.7)
	J	Q	28.0 (28.8)
		R	97.3 (219)
	BL	Q	129 (74.7)
		R	122 (113)
CV	Y	Q	13.3 (20.7)
		R	19.2 (16.1)
	J	Q	9.25 (17.0)
		R	21.0 (79.4)

	BL	Q	19.0 (13.2)
		R	17.4 (14.3)
I	Y	Q	145 (285)
		R	88.4 (17.9)
	J	Q	131 (231)
		R	100 (11.2)
	BL	Q	100 (67.1)
		R	100 (2.53 $\times 10^{-10}$)
c_{tot}	Y	Q	100 (285)
		R	63.2 (24.1)
	J	Q	100 (206)
		R	81.0 (3510)
	BL	Q	36.3 (36.9)
		R	45.7 (66.9)

A IAD VALIDATION

To demonstrate that optical properties output by IAD are reproducible and accurate, the output properties from IAD analysis of measurements of the same BioPixS phantom on three days are shown in Figure A5a and Figure A5b. Whilst there is some variability, the results show that the optical properties returned by this method accurately reflect the ground truth properties.

FIGURE A5 Figure a shows the IAD outputted μ_a and Figure b shows the IAD outputted μ_s' compared to the well-characterised ground truth values for a BioPixS optical phantom measured on three separate days.

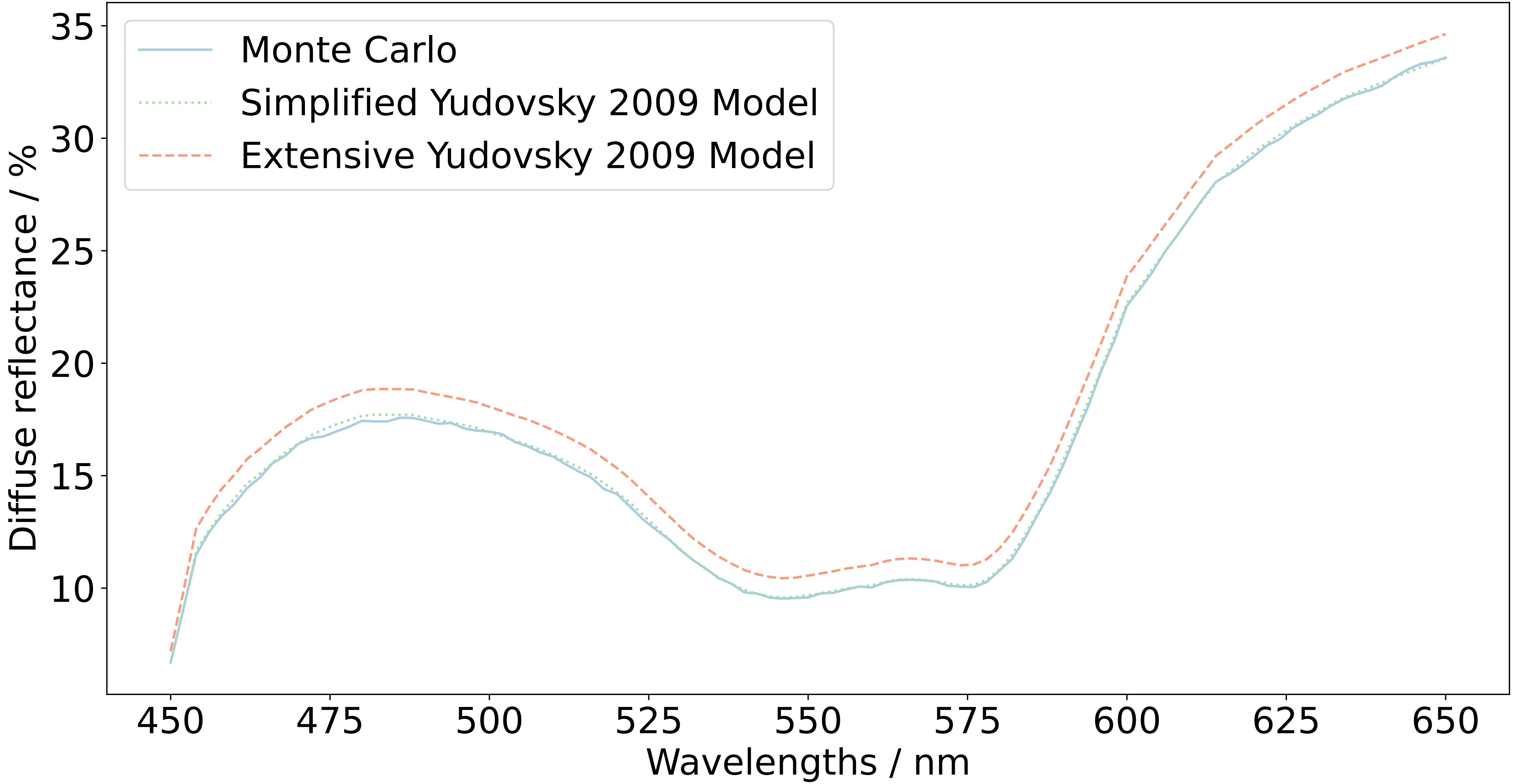
Monte Carlo
Simplified Yudovsky 2009 Model
Extensive Yudovsky 2009 Model
Diffuse reflectance / %
Wavelengths / nm
35
30
25
20
15
10
450
475
500
525
550
575
600
625
650

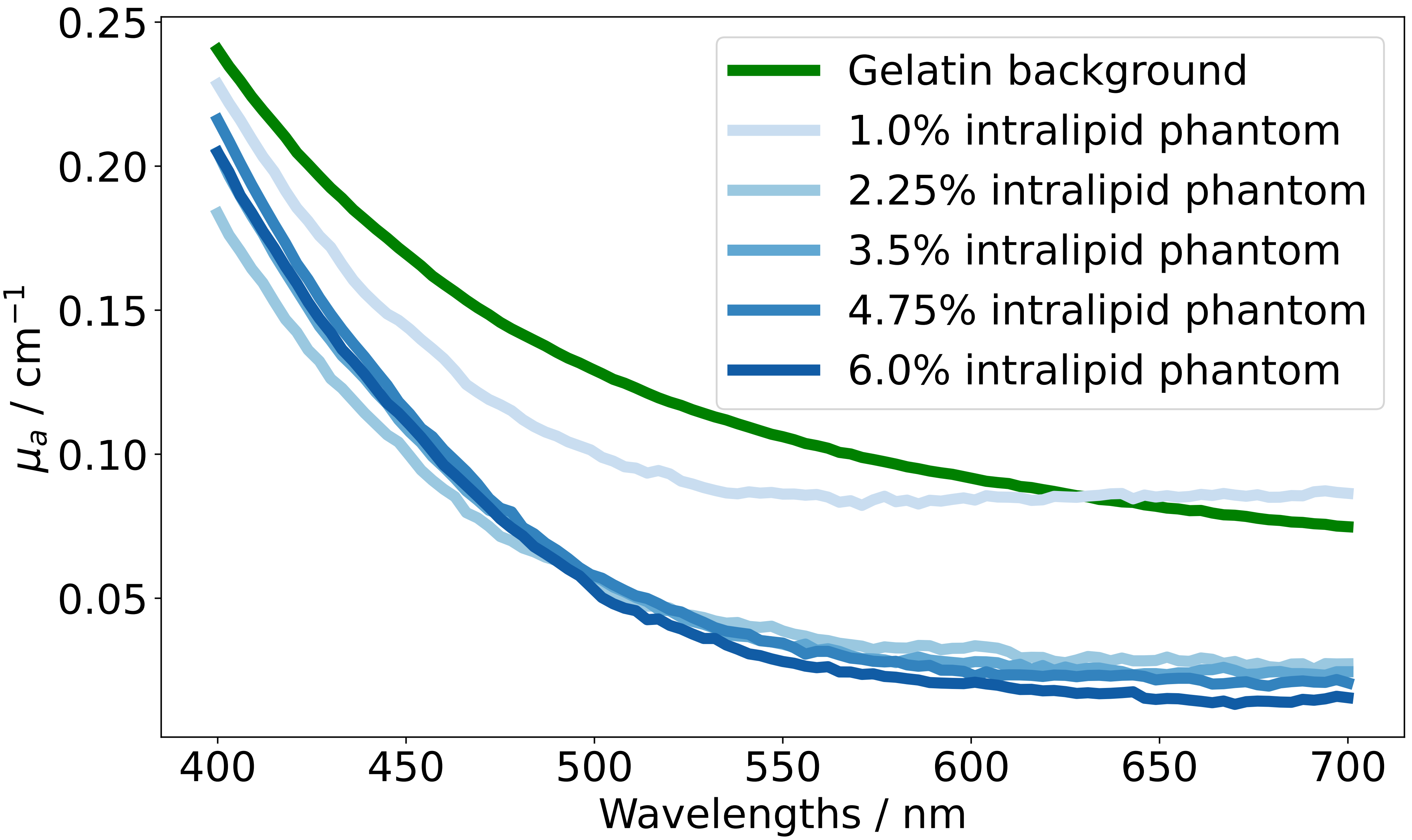
0.25
0.20
0.15
0.10
0.05
μ_a / cm^{-1}
400
450
500
550
600
650
700
Wavelengths / nm
Gelatin background
1.0% intralipid phantom
2.25% intralipid phantom
3.5% intralipid phantom
4.75% intralipid phantom
6.0% intralipid phantom

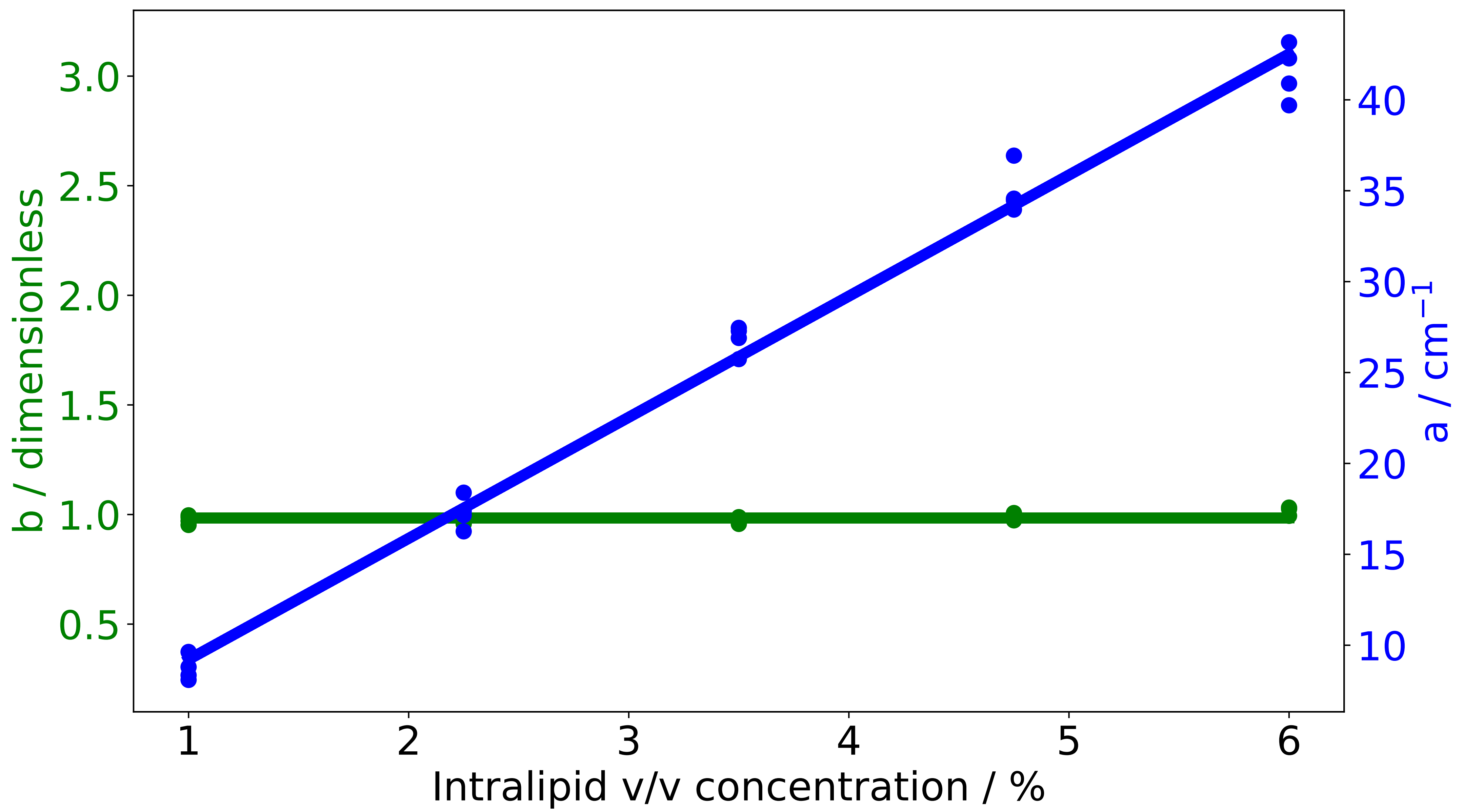

b / dimensionless
3.0
2.5
2.0
1.5
1.0
0.5
a / cm⁻¹
40
35
30
25
20
15
10
1
2
3
4
5
6
Intralipid v/v concentration / %

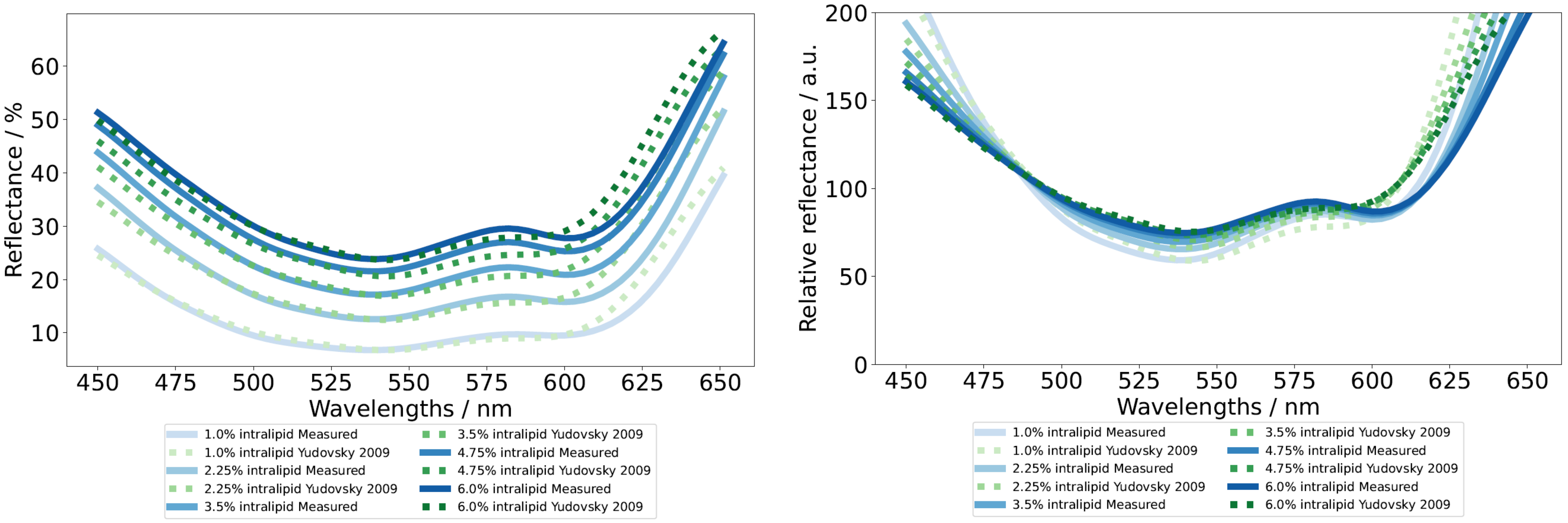
Reflectance / %
Wavelengths / nm
1.0% intralipid Measured
1.0% intralipid Yudovsky 2009
2.25% intralipid Measured
2.25% intralipid Yudovsky 2009
3.5% intralipid Measured
3.5% intralipid Yudovsky 2009
4.75% intralipid Measured
4.75% intralipid Yudovsky 2009
6.0% intralipid Measured
6.0% intralipid Yudovsky 2009
Relative reflectance / a.u.
Wavelengths / nm
1.0% intralipid Measured
1.0% intralipid Yudovsky 2009
2.25% intralipid Measured
2.25% intralipid Yudovsky 2009
3.5% intralipid Measured
3.5% intralipid Yudovsky 2009
4.75% intralipid Measured
4.75% intralipid Yudovsky 2009
6.0% intralipid Measured
6.0% intralipid Yudovsky 2009

(a) (b)

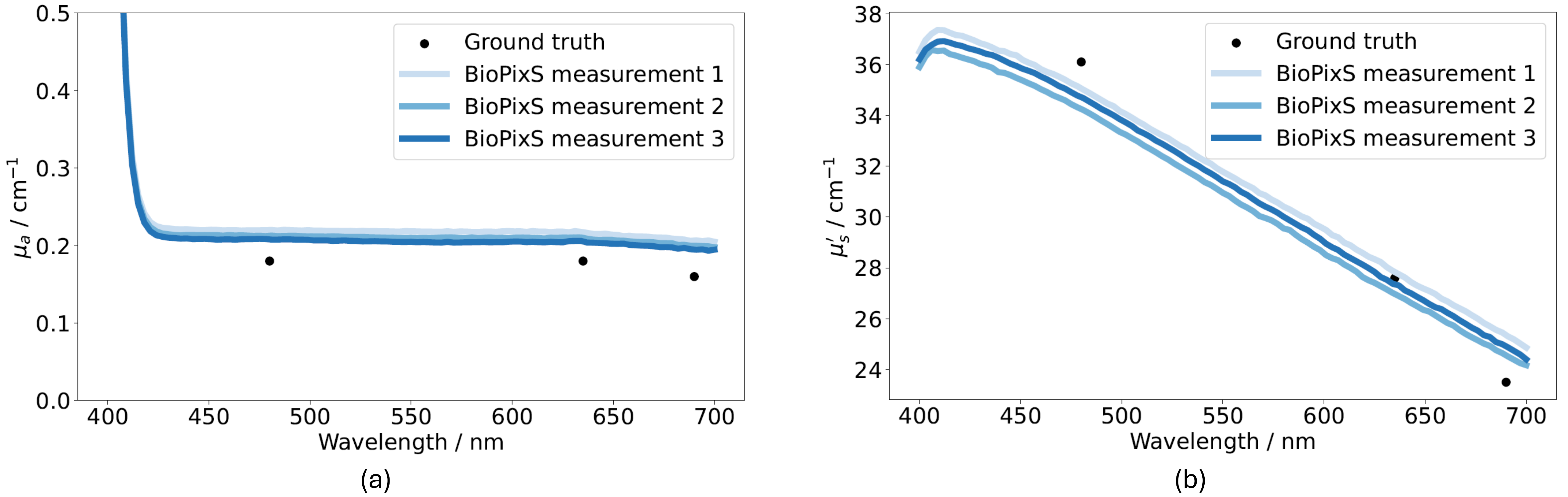

0.5
0.4
0.3
0.2
0.1
0.0
μ_a / cm^{-1}
400
450
500
550
600
650
700
Wavelength / nm
Ground truth
BioPixS measurement 1
BioPixS measurement 2
BioPixS measurement 3
(a)
38
36
34
32
30
28
26
24
μ_s' / cm^{-1}
400
450
500
550
600
650
700
Wavelength / nm
Ground truth
BioPixS measurement 1
BioPixS measurement 2
BioPixS measurement 3
(b)
